# Accuracy of ultrasonic artificial intelligence in diagnosing benign and malignant breast diseases: A protocol for systematic review and meta-analysis

**DOI:** 10.1097/MD.0000000000028289

**Published:** 2021-12-17

**Authors:** Qiyu Liu, Meijing Qu, Lipeng Sun, Hui Wang

**Affiliations:** Ultrasound Department of the First Affiliated Hospital of Dalian Medical University, Dalian City, Liaoning Province, China.

**Keywords:** artificial intelligence, breast diseases, meta-analysis

## Abstract

**Background:**

Artificial intelligence system is a deep learning system based on computer-assisted ultrasonic image diagnosis, which can extract morphological features of breast mass and conduct objective and efficient image analysis, thus automatically intelligent classification of breast mass, avoiding subjective error and improving the accuracy of diagnosis.^[1–2]^ A large number of studies have confirmed that artificial intelligence (AI) has high effectiveness and reliability in the differential diagnosis of benign and malignant breast diseases.^[3–4]^ However, the results of these studies have been contradictory. Therefore, this meta-analysis tested the hypothesis that artificial intelligence system is accurate in distinguishing benign and malignant breast diseases.

**Methods:**

We will search PubMed, Web of Science, Cochrane Library, and Chinese biomedical databases from their inceptions to the November 20, 2021, without language restrictions. Two authors will independently carry out searching literature records, scanning titles and abstracts, full texts, collecting data, and assessing risk of bias. Review Manager 5.2 and Stata14.0 software will be used for data analysis.

**Results:**

This systematic review will determine the accuracy of AI in the differential diagnosis of benign and malignant breast diseases.

**Conclusion:**

Its findings will provide helpful evidence for the accuracy of AI in the differential diagnosis of benign and malignant breast diseases.

**Systematic review registration:**

INPLASY2021110087.

## 1 Introduction

The incidence of breast cancer is increasing year by year, and the trend is younger. In 2020, the number of new cases of breast cancer in the world will reach 2.26 million, becoming the highest incidence of malignant tumor in the world.^[5]^ Ultrasound has become an important means of breast cancer diagnosis because of its advantages of fast and no radioactivity. However, in clinical practice, the ultrasonic manifestations of breast cancer are variable, and the diagnostic level of doctors with different seniority also varies greatly, which are important reasons affecting the diagnostic accuracy of breast cancer.^[6–7]^ Artificial intelligence has provided an important auxiliary diagnostic method for breast ultrasound. Artificial intelligence (AI) decision making comes from the extraction and analysis of morphological and texture features of lesions. Through quantitative analysis of these features, ultrasound diagnosis can be standardized and consistency between observers can be improved.^[8]^ A large number of studies have confirmed that AI has high effectiveness and reliability in the differential diagnosis of benign and malignant breast diseases.

## 2 Materials and methods

This study was conducted in accordance with the PRISMA (Preferred Reporting Items for Systematic Reviews and Meta-Analyses) guidelines and the protocol was registered in the INPLASY (INPLASY2021110087).

### 2.1 Eligibility criteria

#### 2.1.1 Type of study

This study will only include high quality clinical cohort or case control studies.

#### 2.1.2 Type of patients

The patients should be those who had undergone breast diseases.

#### 2.1.3 Intervention and comparison

This study compares AI with pathology for diagnosing breast diseases.

#### 2.1.4 Type of outcomes

The primary outcomes include sensitivity, specifificity, positive and negative likelihood ratio, diagnostic odds ratio, and the area under the curve of the summary receiver operating characteristic.

### 2.2 Search methods

PubMed, Web of Science, Cochrane Library, and Chinese biomedical databases will be searched from their inceptions to the November 20, 2021, without language restrictions. The search strategy for PubMed is shown in Table 1. Other online databases will be used in the same strategy.

**Table 1:** Search strategy sample of pubmed.

Number	Search terms
1	Breast cancer or breast tumors or breast diseases
2	Artificial intelligence or AI or AI-assisted diagnosis system
3	Pathology
4	and 1–3

### 2.3 Data extraction and quality assessment

Two authors will independently select the trials according to the inclusion criteria, and import into Endnote X9 (Thomson Corporation, Stanford, USA). Then remove duplicated or ineligible studies. Screen the titles, abstracts, and full texts of all literature to identify eligible studies. All essential data will be extracted using previously created data collection sheet by 2 independent authors. Discrepancies in data collection between 2 authors will be settled down through discussion with the help of another author. The following data will be extracted from each included research: the first authors surname, publication year, language of publication, study design, sample size, number of lesions, source of the subjects, instrument, “gold standard,” and diagnostic accuracy. The true positives, true negatives, false positives, and false negatives in the fourfold (2 × 2) tables were also collected. Methodological quality was independently assessed by 2 researchers based on the quality assessment of studies of diagnostic accuracy studies (QUADAS) tool. The QUADAS criteria included 14 assessment items. Each of these items was scored as “yes” (2), “no” (0), or “unclear” (1). The QUADAS score ranged from 0 to 28, and a score ≥22 indicated good quality. Any disagreements between 2 investigators will be solved through discussion or consultation by a 3rd investigator.

### 2.4 Statistical analysis

The STATA version 14.0 (Stata Corp, College Station, TX) and Meta-Disc version 1.4 (Universidad Complutense, Madrid, Spain) softwares were used for meta-analysis. We calculated the pooled summary statistics for sensitivity, specificity, positive and negative likelihood ratio, and diagnostic odds ratio with their 95% confidence intervals. The summary receiver operating characteristic curve and corresponding area under the curve were obtained. The threshold effect was assessed using Spearman correlation coefficients. The Cochran's Q-statistic and I test were used to evaluate potential heterogeneity between studies. If significant heterogeneity was detected (Q test *P* < .05 or *I* test > 50%), a random effects model or fixed effects model was used. We also performed sub group and meta-regression analyses to investigate potential sources of heterogeneity. To evaluate the influence of single studies on the overall estimate, a sensitivity analysis was performed. We conducted Begg funnel plots and Egger's linear regression tests to investigate publication bias.

### 2.5 Ethics and dissemination

We will not obtain ethic documents because this study will be conducted based on the data of published literature. We expect to publish this study on a peer-reviewed journal.

## 3 Discussion

By extracting morphological and texture features of benign and malignant lesions, artificial intelligence can help doctors make more detailed analysis of images and provide objective basis for diagnosis. At the same time, AI-assisted diagnosis system can also be used for imaging diagnosis of breast cancer with broad application prospects, including detection of breast lesions, judgment of benign and malignant, pathological classification and prognosis prediction, etc.^[9]^ It has high specificity in differentiating benign and malignant breast lesions, and can become an important auxiliary tool for breast disease diagnosis.

## Author contributions

**Conceptualization:** Lipeng Sun and Hui Wang.

**Data curation:** Meijing Qu and Qiyu Liu.

**Methodology:** Meijing Qu and Qiyu Liu.

**Writing – original draft:** Qiyu Liu.

**Writing – review & editing:** Qiyu Liu and Hui Wang.
